# Panel Data Analysis of Socioeconomic Factors and COVID-19’s Impact on Drinking Habits: Evidence from a Japanese Survey

**DOI:** 10.3390/ijerph22050663

**Published:** 2025-04-23

**Authors:** Makoto Nakakita, Naoki Kubota, Tomoki Toyabe, Sakae Oya, Teruo Nakatsuma

**Affiliations:** 1 Center for Advanced Intelligence Project, RIKEN, Tokyo 103-0027, Japan; 2 Faculty of Economics, Keio University, Tokyo 108-8345, Japan; nk.kubota@keio.jp (N.K.); nakatuma@econ.keio.ac.jp (T.N.); 3 Kokushu Tech Innovation Platform, Tokyo 103-0013, Japan; 4 Faculty of Economics, Kanazawa Gakuin University, Ishikawa 920-1392, Japan; toyabe@kanazawa-gu.ac.jp; 5 Centre for Finance, Technology and Economics at Keio, Keio University, Tokyo 108-8345, Japan; oya-econ@keio.jp

**Keywords:** alcohol use, socioeconomic factor, COVID-19, logit model, Bayesian estimation, Japan

## Abstract

Alcohol use is closely related to health, emotional state, and social behavior. However, no clear consensus exists on how socioeconomic factors influence drinking habits or how the COVID-19 pandemic affected alcohol use. This study examines these relationships in Japan using panel data from a questionnaire survey (2014–2022), consisting of 10,836 responses provided by 1289 respondents, and logistic regression analysis. The results revealed that males, individuals aged 40–69, cigarette smokers, and those who exercise regularly are more likely to drink alcohol, whereas those working at least 2 days per week, earning higher incomes, and in good health are less likely to drink alcohol. The impact of COVID-19 on drinking behavior varied by region, with significant effects observed in specific regions. Additionally, an identified decline in alcohol use since 2016 highlights rising health awareness and shifting values, particularly among younger generations. These findings underscore the strong association between drinking behavior and socioeconomic attributes and emphasize the need to consider regional differences in policy and cultural influences. Overall, this study provides key insights for future research and public health policies on alcohol use in Japan.

## 1. Introduction

Definitions and thresholds for alcohol use vary considerably in the literature. Terms like “moderate drinking”, “excessive drinking”, “unhealthy drinking”, and “heavy drinking” are often used inconsistently and interchangeably, depending on the methodology, population, and regional guidelines. This study provides a foundation for future research and policy discussions related to alcohol use and public health in Japan.

### 1.1. Alcohol Use and Risks

Alcohol use is closely linked to health, emotional state, and social behavior and has long been a major research focus in medicine and psychology. For instance, in psychopharmacology, a subdiscipline of pharmacology, Persson et al. [1] used a double-blind Latin square design to analyze mood ratings by dosage and the correlation between subjective intoxication and mood. Vorobel’ová et al. [2] found that occasional alcohol use was associated with a lower risk of hypertension. Women who reported consuming alcohol occasionally were found to be less susceptible to hypertension than non-drinkers. Meanwhile, an opposite effect was observed in women who consumed alcohol frequently, as they exhibited a higher risk of developing hypertension compared with those who abstained. According to Vorobel’ová et al. [3], alcohol use is significantly associated with night sweats in women. Considering health risks during the COVID-19 pandemic, Ramalho [4] reviewed concerns over rising alcohol use and related harms.

The potential health benefits of moderate alcohol use have been frequently debated in the literature. However, Room et al. [5] emphasized the complexity of alcohol’s health effects, noting that 4% of the global disease burden is attributable to alcohol use. O’Keefe et al. [6] found a J-shaped relationship between alcohol intake and health risks, where moderate consumption appears beneficial but excessive drinking poses major risks. Similar U-shaped patterns of alcohol-related health effects have been widely reported [7,8,9], with Movva and Figueredo [10] noting that the benefits of moderate alcohol use are more prevalent in Western populations. Watanabe et al. [11] linked alcohol use to an increased risk of gastroesophageal reflux disease in males, Otsuka et al. [12] and Nakajima et al. [13] associated it with sleep disorders and reduced sleep quality, and Nomura et al. [14] found that it influences the prevalence of fatty liver disease. Furthermore, Takahashi et al. [15] demonstrated that abstaining from alcohol helps reduce the burden of atrial fibrillation. Miyake et al. [16] reported that alcohol use during pregnancy increases the risk of developmental delays in children, whereas Kiyohara et al. [17] suggested that moderate drinking may lower the risk of systemic lupus erythematosus, a chronic autoimmune disease.

### 1.2. The Social Functions of Alcohol Use

Research has explored alcohol’s effects as well as its relationship with users’ demographic characteristics. Predicting drinking behaviors based on demographic data can help identify unhealthy drinking patterns, such as alcohol abuse, thereby aiding public health from a preventive perspective. Collins [18] indicated that individuals with lower socioeconomic status (SES), particularly those in marginalized communities, face disproportionately negative alcohol-related consequences. Ruhm and Black [19] found that economic recessions are linked to increased heavy drinking. In a study of college students, Wechsler et al. [20] reported that excessive drinkers are more likely to experience alcohol-related problems and that even college attendees who do not drink excessively face an increased risk of secondary harm from others’ excessive consumption. Diniz et al. [21] found that males with low educational attainment and females with high educational attainment show a tendency toward habitual drinking and alcohol abuse. In a U.S.-based study, Rosenquist et al. [22] found that alcohol use behaviors, including heavy drinking and abstinence, can influence not only close friends but also friends of friends. Similarly, in an Australian study, Pettigrew and Roberts [23] reported that alcohol use during social gatherings helps reduce loneliness in old age. Research conducted in Poland by Pavlova et al. [24] indicated that frequent social activities with friends and neighbors correlate with riskier drinking behavior. Among Japanese populations, Kitano et al. [25] compared drinking norms by age and gender between Japanese people in Japan and Japanese Americans residing in Hawaii and California. They found significant regional differences, with Japanese Americans in Hawaii and California displaying similar drinking patterns, whereas Japanese people in Japan differed from both groups.

Okui [26] examined behavioral predictors of moderate and heavy drinking in Japan, finding that moderate alcohol use correlated positively with higher educational status and household income in males and females, whereas heavy drinking was linked to lower educational attainment and smoking in both genders. Using a slightly different dataset and estimation method, Okui [27] further demonstrated that higher educational attainment generally leads to better health outcomes, including reduced psychological distress, better self-rated health, lower smoking rates, and higher cancer screening participation.

### 1.3. Impact of the COVID-19 Pandemic on Drinking Habits

The relationship between drinking habits and individual attributes varies markedly across regions and cultures. During the COVID-19 pandemic, stay-at-home orders in the U.S. and voluntary outing restrictions in Japan likely influenced drinking behaviors. In Japan, the government declared a state of emergency and requested that restaurants suspend alcohol service. According to the Family Income and Expenditure Survey conducted by the Ministry of Internal Affairs and Communications, year-over-year restaurant-based alcohol use decreased by 52.7% in 2020, whereas retail alcohol sales increased by 13.6%. In the remainder of this section, we review prior studies on how the COVID-19 pandemic affected alcohol use worldwide.

Quiroga-Sánchez et al. [28] reported that 22.73% of healthcare workers in a Spanish public hospital increased their alcohol use during the pandemic. Yin et al. [29] used cross-sectional data on middle-aged and older Chinese adults, concluding that leisure physical activity was linked to reduced alcohol use, whereas occupational physical activity correlated with increased drinking. Barbosa et al. [30] reported that excessive and binge drinking rose across all sociodemographic subgroups, considering rates before (February 2020) and after (April 2020) the implementation of pandemic-related stay-at-home orders. In May 2020, Grossman et al. [31] conducted a cross-sectional online survey of US adults aged >21 years, revealing that 60% drank more compared with their prepandemic levels, whereas 13% drank less. Munroe et al. [32] focused on the 13% who reduced their alcohol use, finding that concerns over alcohol’s health risks and competing life demands were key reasons for the decline. In Japan, Nomura et al. [33] assessed the stay-at-home period of the pandemic, reporting that alcohol use was the only major risk factor for suicide-related ideation among university students. Itoshima et al. [34] suggested that the pandemic contributed to increased hospitalizations for alcohol-related liver disease and pancreatitis in Japan. Stickley et al. [35] studied binge drinking in Japan during the pandemic, finding that worsening household finances were associated with current binge drinking and its increase. Additionally, individuals who engaged in binge drinking were significantly less likely to follow COVID-19 preventive measures. Sugaya et al. [36] reported that potential alcoholism and hazardous alcohol use rates were higher than the prepandemic rates recorded in Japan in 2018. Watanabe et al. [37] found that individuals forced to telecommute, despite preferring in-person work, exhibited a significantly greater increase in alcohol use, whereas those who preferred remote work showed no significant change.

The COVID-19 pandemic has also affected the drinking habits of young adults. Falbová et al. [38] found that the prevalence of alcohol use before the pandemic was significantly higher than during the pandemic. Ammar et al. [39] found that the most significant change in consumption habits during the pandemic lockdown was a reduction in binge drinking. According to Pompili et al. [40], living at home may offer protection against alcohol use among young adults. Multi-generational living situations, likely encouraged by pandemic lockdowns, may have contributed to this protection.

### 1.4. Purpose of This Paper

As discussed, there is no complete consensus on the health risks of drinking patterns, the influence of socioeconomic factors on alcohol use, or changes in drinking habits due to the COVID-19 pandemic, as these factors vary by region and culture. Aiming to address this, the current study uses panel data to analyze the relationship between drinking behavior and individual characteristics in Japan and examines how drinking habits changed across demographic groups during the pandemic.

By leveraging panel data, this study accounts for unobservable individual-specific characteristics, enhancing the accuracy of estimations regarding alcohol use patterns. This analytical approach was selected to provide a more accurate understanding of the relationship between various demographic attributes and alcohol use.

## 2. Materials and Methods

### 2.1. Materials

This study uses panel data from a questionnaire survey conducted over 9 years from 2014 to 2022 targeting a Japanese population. The data were provided by the Panel Data Research Center, Institute for Economic Studies, Keio University. The dataset includes 39,250 responses from 5723 respondents across the study period. Data were collected using a self-administered questionnaire. The surveyor distributed the survey form to respondents during an initial visit and subsequently collected the completed forms during a follow-up visit. From 2021 onward, the survey was also made available online for individuals who wished to respond. To account for unobservable factors influencing drinking habits as individual-specific effects, multiple responses from each respondent are necessary for stable estimation. As the logit model used in this study requires individual-specific effects, it cannot estimate whether a respondent’s dependent variable consists entirely of ones or zeros. Thus, respondents with fewer than five responses, those whose answers were all ones or zeros, or those residing outside Japan were excluded from the dataset. After this data processing, the final dataset comprised 10,836 responses from 1289 respondents. Missing data due to nonresponses or input errors were imputed using the k-nearest neighbors (kNN) algorithm based on machine learning. The kNN method is commonly used for data completion in various fields, including healthcare, e.g., [41,42,43]. Although the survey covers a broad range of topics, explanatory variables were selected based on the study’s objective and prior literature, as shown in Table 1, Table 2, Table 3 and Table 4. The dependent variable was set to 1 if respondents reported drinking alcohol at least once per month and 0 if they reported no alcohol use.

Considering potential structural change after 2020 due to the COVID-19 pandemic, variables with overlines were created using data only from 2021 and 2022. Notably, the annual survey was conducted in February, meaning that the data for a given year (e.g., 2021) reflect the situation from the prior year (e.g., 2020). Therefore, the data from 2021 and 2022 capture conditions during the COVID-19 pandemic, which began in 2020.

### 2.2. Methods

This study estimates the impact of residential area, economic status, and life satisfaction on drinking habits by constructing a binary logit model. The binary logit model is a type of binary choice model where the dependent variable can only take on the values 0 or 1. Logit models are often applied in the fields of probability theory and statistics and are widely used to describe the probability of an event occurring. As individuals likely have unobservable characteristics related to their drinking habits, treating these as individual-specific effects allows for unbiased estimates of the influence of socioeconomic attributes. Estimation was performed using Bayesian analysis using Markov chain Monte Carlo methods, specifically the ancillary–sufficiency interweaving strategy proposed by Yu and Meng [44]. For further details on the estimation methodology, refer to Nakakita and Nakatsuma [45].

## 3. Results

Table 5 presents the results of logistic regression models examining the relationship between drinking habits and socioeconomic characteristics, as well as the impact of the COVID-19 pandemic, in Japan. Two models were estimated: one including the impact of the pandemic and one without it.

In both models, males, individuals aged 40–69, cigarette smokers, and those taking regular exercise were more likely to consume alcohol. Conversely, individuals who worked at least 2 days per week, had higher incomes, or reported better health were less likely to drink.

Regarding the effect of the COVID-19 pandemic, the structural change variables for the pandemic period (2021 and 2022) were significant only among individuals in the Tohoku and Chubu regions. No significant effects were observed in other regions.

Additionally, a clear temporal trend was identified: from 2016 to 2022, the overall tendency to consume alcohol declined.

## 4. Discussion

This study examined the relationship between drinking habits and socioeconomic characteristics in Japan, along with the impact of the COVID-19 pandemic, using logistic regression analysis. The findings indicated that males, individuals aged 40–69, cigarette smokers, and those with regular exercise habits were more likely to consume alcohol. Conversely, individuals who worked at least 2 days per week, had high incomes, and reported good health were less likely to drink. This section explores these results in greater depth, examining the individual and social factors influencing drinking behaviors.

### 4.1. Factors Influencing Drinking Behavior

This study’s results confirm that drinking alcohol is more prevalent in males than in females, which may reflect cultural norms in Japan, which makes alcohol use more socially acceptable for men [25]. Furthermore, this study revealed that individuals aged 40–69 have a higher propensity for alcohol use. These findings align with a survey by the Japanese Ministry of Health, Labor and Welfare, which reported that the habitual drinking rates in men aged 40–69 exceeded the national average of 33.9%. In contrast, younger generations exhibited lower drinking tendencies, supporting the widely recognized trend of declining alcohol use among young people. Previous surveys have also indicated that habitual drinking rates among individuals aged 20–39 are markedly lower than the overall average, suggesting that changing lifestyles and growing health consciousness among younger populations may be key contributing factors. Additionally, cigarette smokers were found to have a higher likelihood of alcohol use. This finding is consistent with those of Nakamura et al. [46], who reported a dose–response relationship in which increased cigarette smoking was associated with increased alcohol intake. Interestingly, individuals who engaged in regular exercise were also more likely to consume alcohol. This contrasts with the findings of Yin et al. [29], who found that leisure-time physical activity was linked to reduced alcohol use, suggesting that exercise and drinking behaviors may interact differently across populations and cultural contexts.

Individuals who work full-time demonstrated a lower tendency to drink. This suggests that work-related commitments may limit opportunities for alcohol use. However, this finding differs from that of Morikawa et al. [47], who found no significant correlation between work demands and alcohol use. The present study found that individuals with higher incomes in Japan were less likely to consume alcohol, aligning with the findings by Okui [26], who identified a correlation between high household income and moderate drinking. However, this finding conflicts with a study from Wisconsin, USA, where [48] indicated that higher income levels were associated with increased alcohol use, as well as research by Collins [18], who showed that alcohol use levels among individuals with higher SES were equal to or higher than those among individuals with lower SES. This discrepancy may indicate that in Japan, people with greater financial resources are more inclined to regulate their alcohol intake. Finally, individuals reporting good health exhibited lower alcohol use tendencies, a result aligning with the findings by Tsugane et al. [49], who found that excessive alcohol use markedly increases health risks. This suggests that health-conscious individuals may actively limit their drinking to maintain overall well-being.

### 4.2. Impact of COVID-19

In this study, the analysis that accounted for COVID-19 revealed significant positive effects on drinking tendencies only in the Tohoku and Chubu regions. However, all other variables showed no significant structural changes due to COVID-19. There are several possible reasons for this result. However, as can be seen in the Ministry of Health, Labor and Welfare’s COVID-19 data (Ministry of Health, Labor and Welfare, “Visualizing the data: information on COVID-19 infections” https://covid19.mhlw.go.jp/ (accessed on 5 April 2025)) regarding the number of infected persons by prefecture, the Tohoku and Chubu regions—including the prefectures of Iwate, Fukushima, Niigata, and Nagano—had fewer COVID-19 cases compared with the national average, which may have contributed to these results. The widespread restrictions on restaurant operations and government encouragement to stay at home during the pandemic may have contributed to the observed regional differences. In areas where drinking increased, the closure of bars and restaurants may have led individuals to consume more alcohol at home. Conversely, in regions where COVID-19 had no significant impact on drinking behavior, the rise in home drinking due to remote work may have balanced out the decline in social drinking opportunities.

### 4.3. Decline in Alcohol Use Since 2016

This study’s results highlight a continuous decline in alcohol use across Japan from 2016 to 2022 (National Tax Agency, “Tax Statistics”, https://www.nta.go.jp/publication/statistics/kokuzeicho/tokei.htm (accessed on 5 April 2025)). This long-term shift appears to be driven by broader social and cultural factors rather than the immediate effects of the COVID-19 pandemic. In recent years, increased health awareness, facilitated by social media and other forms of mass media, has contributed to a growing preference for reducing or abstaining from alcohol use. This trend is particularly pronounced among younger generations, who are more exposed to health-conscious messaging and lifestyle choices (Ministry of Health, Labor and Welfare, “Drinking habits by sex and age group”, https://www.mhlw.go.jp/topics/bukyoku/kenkou/alcohol/siryo/insyu03.html (accessed on 5 April 2025)). Notably, the rise in alcohol-free events at corporate gatherings and university orientation events further reflects this societal shift.

### 4.4. Significance and Limitations

This study makes several important contributions to understanding drinking behavior in Japan. First, it empirically demonstrates the relationship between alcohol use and socioeconomic factors, showing that drinking tendencies are associated with variables such as gender, age, smoking status, exercise habits, employment, income, and overall health condition. Second, it provides a quantitative analysis of regional variations in the impact of COVID-19 on drinking habits, highlighting significant effects in the Tohoku and Chubu regions, but not elsewhere. Finally, it identifies a long-term decline in drinking habits since 2016, suggesting that increased health consciousness and shifting values, particularly among younger generations, are key drivers of this trend. These findings suggest that changes in drinking behavior are not merely temporary reactions to the COVID-19 pandemic but rather part of a broader and ongoing societal transformation.

Despite its contributions, this study has several limitations that should be addressed in future research. First, it lacks a detailed analysis of drinking frequency and alcohol use volume. Specifically, drinking behavior was classified as a binary variable (drinker vs. nondrinker); thus, drinking frequency and volume consumed were not considered. Future studies should incorporate more granular measures, such as daily, weekly, or monthly consumption, as well as moderate vs. heavy drinking, to provide a more comprehensive analysis of drinking in Japan. Second, the reliance on self-reported survey data introduces the possibility of social desirability bias, where respondents may underreport or overreport their drinking habits to present themselves in a favorable light. Future research should incorporate objective data sources, such as alcohol sales records or medical examination data, to enhance reliability.

## 5. Conclusions

This study examined the relationship between drinking habits and socioeconomic factors in Japan, as well as the impact of the COVID-19 pandemic, using panel data and logistic regression analysis. The findings indicate that males, middle-aged and older individuals (40–69 years), cigarette smokers, and those with regular exercise habits are more likely to consume alcohol. In contrast, individuals who work at least 2 days per week, those with higher incomes, and those in good health exhibit lower drinking tendencies. The study also revealed regional differences regarding the impact of COVID-19 on drinking, with notable effects observed only in the Tohoku and Chubu regions. Additionally, a long-term decline in alcohol use across Japan since 2016 was identified, with increased health consciousness and shifting social values, especially among younger generations, suggested as contributing factors.

These findings have several important implications. First, they provide empirical evidence of how drinking habits are influenced by socioeconomic attributes, contributing to a deeper understanding of alcohol use patterns in Japan. Second, the regional variations in the impact of COVID-19 highlight the need to consider local cultural and policy factors when assessing changes in drinking behavior. Third, the long-term decline in drinking tendencies suggests that changes in social norms and public health awareness are influencing alcohol use beyond the immediate effects of the pandemic. These insights may be valuable for policymakers seeking to develop effective public health strategies in Japan. Since drinking habits vary across demographic groups, this study will help them implement campaigns that raise awareness about issues various demographics may face from alcohol abuse, or support groups tailored to people based on age, gender, etc. 

## Figures and Tables

**Table 1:** Descriptive statistics for explained and dummy variables (entire period).

		Frequency		Mean	
Variable	Description	Yes	No	Yes	No
Drink	Drink (default: No)				
*y*	Yes	5872	4973	1	0
Region	Region in which respondent resides (default: Kanto)				
$x_{area,1}$	Hokkaido	485	10,351	0.569	0.540
$x_{area,2}$	Tohoku	723	10,113	0.487	0.545
$x_{area,3}$	Chubu	1936	8900	0.523	0.545
$x_{area,4}$	Kinki	2055	8781	0.522	0.546
$x_{area,5}$	Chugoku	614	10,222	0.547	0.541
$x_{area,6}$	Shikoku	314	10,522	0.573	0.540
$x_{area,7}$	Kyushu	1167	9669	0.577	0.537
Size of city	Size of city				
$x_{city,1}$	Other cities	6667	4169	0.541	0.542
$x_{city,2}$	Towns and villages	952	9884	0.539	0.541
Year	(default: 2014)				
$x_{1,1}$	2015	1287	9549	0.667	0.524
$x_{1,2}$	2016	1283	9553	0.613	0.532
$x_{1,3}$	2017	1283	9553	0.607	0.532
$x_{1,4}$	2018	1281	9555	0.540	0.541
$x_{1,5}$	2019	1214	9622	0.505	0.546
$x_{1,6}$	2020	1150	9686	0.450	0.552
$x_{1,7}$	2021	1047	9789	0.402	0.556
$x_{1,8}$	2022	1006	9830	0.346	0.561
Spouse	Having a spouse (default: No)				
$x_{1}$	Yes	8067	2769	0.548	0.523
Sex	(default: Female)				
$x_{2}$	Male	4410	6426	0.578	0.516
Age	(default: –39)				
$x_{3,1}$	40–49	2423	8413	0.552	0.538
$x_{3,2}$	50–59	2322	8514	0.542	0.541
$x_{3,3}$	60–69	2033	8803	0.567	0.535
$x_{3,4}$	70–	2210	8626	0.489	0.555
Work	(default: Mostly worked)				
$x_{4,1}$	Worked while mostly attending school or keeping house	1611	9225	0.534	0.542
$x_{4,2}$	Did not perform any paid work	3487	7349	0.494	0.563
Smoking	(default: No)				
$x_{8}$	Yes	1715	9121	0.607	0.529
Medical examination	(default: No exam or screening)				
$x_{9,1}$	Received and problems noted	3173	7663	0.567	0.531
$x_{9,2}$	Received and no problems noted	3439	7397	0.547	0.539
Regular exercise	(default: No)				
$x_{10}$	Yes	4659	6177	0.552	0.533

**Table 2:** Descriptive statistics for quantitative variables (entire period).

Variable	Description	Mean	SD	Median	Max	Min
Income	Income from main job in the past year					
$x_{5}$	log (income + 1)	3.702	2.627	4.710	8.007	0
Happiness	Feeling complete happiness in the present year					
$x_{6}$	(−5–5)	1.049	2.176	1	5	−5
Health	Subjective health status					
$x_{7}$	(Good (−2)–Bad (2))	−0.303	0.948	0	2	−2
Satisfaction level	Life overall					
$x_{11}$	(Dissatisfied (−5)–Satisfied (5))	0.891	2.114	1	5	−5

Responses regarding satisfaction are centered, with “neither satisfied nor dissatisfied” being zero. Satisfaction is measured on a scale from dissatisfied (−5) to satisfied (5).

**Table 3:** Descriptive statistics for explained and dummy variables (COVID-19 period).

		Frequency		Mean	
Variable	Description	Ye	No	Yes	No
Drink	Drink (default: No)				
$\bar{y}$	Yes	769	1284	1	0
Region	Region in which respondent resides (default: Kanto)				
${\bar{x}}_{area,1}$	Hokkaido	88	1965	0.409	0.343
${\bar{x}}_{area,2}$	Tohoku	144	1909	0.389	0.373
${\bar{x}}_{area,3}$	Chubu	357	1696	0.398	0.370
${\bar{x}}_{area,4}$	Kinki	400	1653	0.355	0.379
${\bar{x}}_{area,5}$	Chugoku	120	1933	0.375	0.375
${\bar{x}}_{area,6}$	Shikoku	57	1996	0.404	0.373
${\bar{x}}_{area,7}$	Kyushu	224	1829	0.379	0.374
Size of city	Size of city				
${\bar{x}}_{city,1}$	Other cities	1289	764	0.370	0.382
${\bar{x}}_{city,2}$	Towns and villages	175	1878	0.389	0.373
Year	(default: 2014)				
$x_{1,7}$	2021	1047	9789	0.402	0.556
$x_{1,8}$	2022	1006	9830	0.346	0.561
Spouse	Having a spouse (default: No)				
${\bar{x}}_{1}$	Yes	1515	538	0.389	0.333
Sex	(default: Female)				
${\bar{x}}_{2}$	Male	824	1229	0.380	0.371
Age	(default: –39)				
${\bar{x}}_{3,1}$	40–49	463	1590	0.406	0.365
${\bar{x}}_{3,2}$	50–59	481	1572	0.383	0.372
${\bar{x}}_{3,3}$	60–69	371	1682	0.358	0.378
${\bar{x}}_{3,4}$	70–	542	1511	0.347	0.385
Work	(default: Mostly worked)				
${\bar{x}}_{4,1}$	Worked while mostly attending school or keeping house	301	1752	0.369	0.376
${\bar{x}}_{4,2}$	Did not perform any paid work	667	1386	0.357	0.383
Smoking	(default: No)				
${\bar{x}}_{8}$	Yes	262	1791	0.389	0.513
Medical examination	(default: No exam or screening)				
${\bar{x}}_{9,1}$	Received and problems noted	545	1508	0.433	0.353
${\bar{x}}_{9,2}$	Received and no problems noted	698	1355	0.370	0.377
Regular exercise	(default: No)				
${\bar{x}}_{10}$	Yes	900	1153	0.378	0.372

**Table 4:** Descriptive statistics for quantitative variables (COVID-19 period).

Variable	Description	Mean	SD	Median	Max	Min
Income	Income from main job in the past year					
${\bar{x}}_{5}$	log(income + 1)	3.704	2.650	4.727	7.784	0
Happiness	Feeling complete happiness in the past year					
${\bar{x}}_{6}$	(−5–5)	0.860	2.156	1	5	−5
Health	Subjective health status					
${\bar{x}}_{7}$	(Good (−2)–Bad (2))	−0.237	0.955	0	2	−2
Satisfaction level	Life overall					
${\bar{x}}_{11}$	(Dissatisfied (−5)–Satisfied (5))	1.045	2.059	1	5	−5

Responses regarding satisfaction are centered, with “neither satisfied nor dissatisfied” being zero. Satisfaction is measured on a scale from dissatisfied (−5) to satisfied (5).

**Table 5:** Posterior means and standard deviations of Model A (all respondents). Values shown in bold are statistically significant at the 5% level.

β	COVID-19 Mean	COVID-19 SD	Baseline Mean	Baseline SD
$\beta_{area,1}$	0.091	0.229	0.125	0.222
$\beta_{area,2}$	−0.468	0.197	−0.343	0.192
$\beta_{area,3}$	−0.28	0.133	−0.196	0.128
$\beta_{area,4}$	−0.154	0.131	−0.128	0.126
$\beta_{area,5}$	−0.075	0.208	−0.031	0.196
$\beta_{area,6}$	−0.017	0.279	0.014	0.266
$\beta_{area,7}$	0.132	0.168	0.135	0.155
$\beta_{city,1}$	0.059	0.101	0.037	0.094
$\beta_{city,2}$	0.06	0.178	0.057	0.169
$\beta_{2015}$	0.056	0.098	0.055	0.096
$\beta_{2016}$	−0.288	0.096	−0.283	0.096
$\beta_{2017}$	−0.306	0.098	−0.303	0.096
$\beta_{2018}$	−0.674	0.097	−0.668	0.096
$\beta_{2019}$	−0.908	0.099	−0.9	0.098
$\beta_{2020}$	−1.245	0.101	−1.234	0.1
$\beta_{2021}$	−1.456	0.563	−1.788	0.357
$\beta_{2022}$	−1.762	0.566	−2.09	0.357
$\beta_{1}$	−0.026	0.101	0.006	0.095
$\beta_{2}$	0.275	0.102	0.223	0.097
$\beta_{3,1}$	0.224	0.112	0.25	0.104
$\beta_{3,2}$	0.305	0.126	0.315	0.121
$\beta_{3,3}$	0.425	0.134	0.369	0.13
$\beta_{3,4}$	0.273	0.148	0.227	0.14
$\beta_{4,1}$	−0.221	0.111	−0.219	0.105
$\beta_{4,2}$	−1.099	0.168	−1.062	0.156
$\beta_{5}$	−0.104	0.031	−0.109	0.028
$\beta_{6}$	−0.002	0.018	−0.0	0.016
$\beta_{7}$	−0.132	0.039	−0.141	0.036
$\beta_{8}$	0.495	0.111	0.477	0.107
$\beta_{9,1}$	0.113	0.081	0.158	0.076
$\beta_{9,2}$	0.138	0.082	0.154	0.076
$\beta_{10}$	0.149	0.068	0.143	0.062
$\beta_{11}$	0.021	0.02	0.02	0.018
$\beta_{12,1}$	0.101	0.241	0.181	0.239
$\beta_{12,2}$	0.034	0.16	0.038	0.16
$\beta_{12,3}$	0.141	0.349	0.159	0.351
$\beta_{13,1}$	0.008	0.189	0.07	0.184
$\beta_{13,2}$	0.143	0.143	0.16	0.137
$\beta_{14,1}$	0.457	0.458	0.539	0.449
$\beta_{14,2}$	−0.2	0.14	−0.166	0.137
$\beta_{15,1}$	−0.065	0.207	−0.006	0.203
$\beta_{15,2}$	0.025	0.25	0.035	0.244
$\beta_{16,2}$	0.574	0.314	0.559	0.31
${\bar{\beta }}_{area,1}$	0.199	0.322		
${\bar{\beta }}_{area,2}$	0.713	0.264		
${\bar{\beta }}_{area,3}$	0.473	0.183		
${\bar{\beta }}_{area,4}$	−0.136	0.18		
${\bar{\beta }}_{area,5}$	0.148	0.283		
${\bar{\beta }}_{area,6}$	0.247	0.385		
${\bar{\beta }}_{area,7}$	0.028	0.221		
${\bar{\beta }}_{city,1}$	−0.127	0.144		
${\bar{\beta }}_{city,2}$	−0.037	0.249		
${\bar{\beta }}_{1}$	0.213	0.151		
${\bar{\beta }}_{2}$	−0.273	0.146		
${\bar{\beta }}_{3,1}$	−0.112	0.234		
${\bar{\beta }}_{3,2}$	−0.195	0.237		
${\bar{\beta }}_{3,3}$	−0.462	0.255		
${\bar{\beta }}_{3,4}$	−0.439	0.26		
${\bar{\beta }}_{4,1}$	0.042	0.216		
${\bar{\beta }}_{4,2}$	0.187	0.372		
${\bar{\beta }}_{5}$	−0.046	0.066		
${\bar{\beta }}_{6}$	0.012	0.039		
${\bar{\beta }}_{7}$	−0.048	0.079		
${\bar{\beta }}_{8}$	−0.169	0.191		
${\bar{\beta }}_{9,1}$	0.237	0.169		
${\bar{\beta }}_{9,2}$	0.052	0.158		
${\bar{\beta }}_{10}$	−0.034	0.13		
${\bar{\beta }}_{11}$	−0.009	0.042		
$\mu_{\alpha }$	0.672	0.396	0.668	0.379
$\sigma_{\alpha }$	1.786	0.114	1.769	0.112
WAIC	3570.2		3745.8	

## Data Availability

Restrictions apply to the availability of the data. Data were obtained from Keio University and are available at https://www.pdrc.keio.ac.jp/en/ (accessed on 7 November 2024) with the permission of Keio University.

## Abbreviations

The following abbreviations are used in this manuscript:

ASIS	ancillarity–sufficiency interweaving strategy
COVID-19	Coronavirus disease 2019
MCMC	Markov chain Monte Carlo
SES	socioeconomic status
kNN	k-nearest neighbors
